# Genome Sequences of Community Carriage Strains of Staphylococcus aureus from Yuma, Arizona

**DOI:** 10.1128/MRA.00449-21

**Published:** 2021-09-16

**Authors:** Talima Pearson, Crystal Hepp, Robert T. Trotter, Mimi Mbegbu, Benjamin Russakoff, David Panisello Yagüe, Colin Wood, Kara Tucker-Morgan, Kathya Ceniceros, Cristina Padilla, Shari Kyman, Francisco Villa

**Affiliations:** a Pathogen and Microbiome Institute, Northern Arizona University, Flagstaff, Arizona, USA; b Center for Health Equity Research, Northern Arizona University, Flagstaff, Arizona, USA; c School of Informatics, Computing, and Cyber Systems, Northern Arizona University, Flagstaff, Arizona, USA; d Department of Anthropology, Northern Arizona University, Flagstaff, Arizona, USA; e Northern Arizona University—Yuma, Yuma, Arizona, USA; University of Rochester School of Medicine and Dentistry

## Abstract

Staphylococcus aureus exists as a pathogen and commensal. Individuals with asymptomatic carriage serve as a reservoir for transmission and are at increased risk of infecting themselves. In order to characterize the genomic diversity of S. aureus circulating in the community, we sequenced 166 genomes collected from individuals in Yuma, AZ.

## ANNOUNCEMENT

Staphylococcus aureus is a Gram-positive bacterium that lives in close association with humans as both a commensal and pathogen (1). S. aureus is a common cause of skin, soft tissue, bone, joint, respiratory, and endovascular infections that occur when these bacteria penetrate the outer layers of skin or mucosa (2–7). Methicillin-resistant S. aureus (MRSA) and methicillin-susceptible S. aureus (MSSA) infections have historically been considered a health care-acquired (HA) phenomenon; however, new lineages, and even traditionally HA strains, are circulating at an increased rate in the community (1, 8–11). Controlling this MSSA/MRSA epidemic is particularly challenging because of a large community-based reservoir; about 19 to 55% of the general population are colonized in the nares by S. aureus, with 1.5% being carriers of MRSA (3, 12). Colonization usually precedes infection and is therefore the major risk factor for disease (2, 5, 13–16).

Ethnic minorities and those with less education are typically at greater risk for a variety of infectious diseases. National data suggest that S. aureus carriage in Hispanics is not significantly different than for non-Hispanic whites, but people born in Mexico and those with higher levels of education have a lower risk of colonization (17). Our data from near the binational U.S./Mexico border in Yuma, AZ, show very high carriage rates, no educational or other socioeconomic gradient on S. aureus colonization, but a nominally lower colonization rate in Hispanics compared to non-Hispanics (18). To more fully understand the genomic aspects of community carriage in this predominantly Hispanic population, we collected and sequenced strains of S. aureus from participants in Yuma, AZ (19). All samples were collected as part of project 1116783, approved by the Northern Arizona University Institutional Review Board. Here, we present the genome sequences of isolates collected from March through June 2019. We used BBL CultureSwab swabs to sample three body sites (the anterior nares, throat, and palm) of each participant, cultured samples on CHROMagar Staph aureus medium, and selected mauve colonies for DNA extraction and sequencing (19). For DNA extraction, we used Qiagen DNeasy blood and tissue kits with added lysostaphin at a final concentration of 0.75 U/μl and an incubation time of 1 h. We constructed libraries using 500 to 1,000 ng genomic DNA (gDNA) with the Kapa HyperPrep kit (Roche Diagnostics) and conducted mechanical shearing on the QSonica Q800R3 sonicator. Double-sided size selection was performed using AMPure XP paramagnetic beads (Beckman Coulter) for 600- to 700-bp fragments. The libraries were pooled equimolar using the KAPA library quantification kit (Roche Diagnostics). DNA from 166 colonies was sequenced on an Illumina NextSeq 500 instrument using a 150-cycle 500/550 high-output kit v2.5. Assembly, error correction, and quality trimming were performed using SPAdes v2.0 (20), and contigs shorter than 500 bp were removed. The average genome coverage was calculated using BBMap (21), and the Prokaryotic Genome Annotation Pipeline (PGAP) was used for annotation (22).

The multilocus sequence type and staphylococcal cassette chromosome *mec* (SCC*mec*) type were determined for each genome using MLST v2.0 and SCCmecFinder v1.2 (23). Default settings were used for all software unless otherwise specified. The genome metrics and isolation source information are provided in Table 1.

**TABLE 1:** Characteristics and accession numbers for 166 S. aureus genomes

Isolate^*a*^	SCC*mec*	ST^*b*^	*N*_50_ (bp)	No. of contigs	Size (bp)	Coverage (×)	GC content (%)	No. of reads	GenBank accession no.
2a2005n1	MSSA	30	62,943	126	2,758,377	123.726	0.3272	4,584,868	JAGRXT000000000
2a2005t1	MSSA	30	62,943	118	2,756,207	112.034	0.3272	4,167,611	JAGRXS000000000
2a2009n1	MSSA	5	187,277	72	2,788,303	670.834	0.3274	12,611,705	JAGRXR000000000
2a2009t1	MSSA	5	53,193	100	2,760,346	41.312	0.3326	2,647,703	JAGRXQ000000000
2a2010t1	MSSA	188	80,305	73	2,721,189	150.941	0.3268	5,538,066	JAGRXP000000000
2a2015t1	MSSA	5^*d*^	81,690	79	2,718,012	117.74	0.327	4,263,633	JAGRXO000000000
2a2017n1	MSSA	30	65,351	103	2,741,214	118.954	0.3271	4,373,292	JAGRXN000000000
2a2023n1	MSSA	30	58,969	111	2,794,809	115.499	0.3272	4,465,014	JAGRXM000000000
2a2024n1	MSSA	30	61,394	104	2,782,339	55.534	0.3284	2,290,514	JAGRXL000000000
2a2024p1	MSSA	30	54,206	111	2,781,358	38.434	0.3305	2,178,584	JAGRXK000000000
2a2024t1	MSSA	30	51,415	116	2,780,566	28.615	0.3342	1,220,699	JAGRXJ000000000
2a2026n1	MSSA	8	96,971	75	2,747,384	150.708	0.3267	5,577,335	JAGRXI000000000
2a2027n1	MSSA	30	65,368	113	2,743,712	131.177	0.3271	4,863,836	JAGRXH000000000
2a2027t1	MSSA	30	66,081	114	2,744,916	158.258	0.3272	5,797,055	JAGRXG000000000
2a2029n1	MSSA	398	71,211	74	2,662,941	127.571	0.3282	4,584,259	JAGRXF000000000
2a2031t1	MSSA	U	14,768	482	2,865,289	115.47	0.3271	4,759,290	JAGRXE000000000
2a2036t1	MSSA	U	79,805	81	2,684,634	32.237	0.3293	1,504,102	JAGRXD000000000
2a2043n1	MSSA	15	51,742	103	2,706,404	56.862	0.3276	2,269,209	JAGRXC000000000
2a2043t1	MSSA	15	46,199	106	2,656,310	33.421	0.3308	1,302,761	JAGRXB000000000
2a2048n1	MSSA	30	61,993	125	2,817,267	80.866	0.3277	3,425,204	JAGRXA000000000
2a2048t1	MSSA	87	54,033	99	2,678,932	56.111	0.3279	2,009,529	JAGRWZ000000000
2a2049n1	IVg(2B)	5	67,593	97	2,836,262	132.995	0.3278	5,038,467	JAGRWY000000000
2a2049t1	IVg(2B)	5	67,509	93	2,836,243	144.04	0.3279	5,439,365	JAGRWX000000000
2a2050n1	MSSA	582	50,864	114	2,683,760	44.349	0.3278	1,619,432	JAGRWW000000000
2a2050p1	MSSA	582	61,484	95	2,687,584	127.061	0.3275	4,595,761	JAGRWV000000000
2a2051n1	MSSA	582	94,535	54	2,680,589	893.164	0.328	15,895,551	JAGRWU000000000
2a2055t1	MSSA	45	75,685	65	2,696,835	133.122	0.3285	4,791,738	JAGRWT000000000
2a2071t1	MSSA	5	61,800	89	2,721,622	39.798	0.3283	1,495,182	JAGRWS000000000
2a2074n1	MSSA	6	59,828	96	2,746,279	131.777	0.3276	4,839,566	JAGRWR000000000
2a2074t1	MSSA	6	44,465	106	2,745,419	34.919	0.3276	1,474,447	JAGRWQ000000000
2a3002n1	MSSA	8^*d*^	87,379	84	2,752,739	123.692	0.327	4,624,050	JAGRWP000000000
2a3005n1	MSSA	97	47,296	101	2,749,588	122.317	0.3272	4,598,588	JAGRWO000000000
2a3007t1	MSSA	188	73,083	83	2,701,956	127.269	0.3269	4,636,473	JAGRWN000000000
2a3015n1	MSSA	45	268,476	26	2,746,283	691.144	0.328	12,635,772	JAGRWM000000000
2a3015p1	MSSA	45	67,650	73	2,735,213	152.045	0.3289	5,723,059	JAGRWL000000000
2a3017n1	MSSA	30	65,233	109	2,764,946	130.212	0.3274	4,843,563	JAGRWK000000000
2a3017t1	MSSA	30	66,082	108	2,764,440	114.524	0.3274	4,269,372	JAGRWJ000000000
2a3037t1	MSSA	109	53,182	86	2,717,649	145.335	0.3269	5,265,909	JAGRWI000000000
2a3067n1	MSSA	15	50,823	99	2,684,656	103.896	0.3274	3,786,964	JAGRWH000000000
2a4019t1	MSSA	5	61,501	83	2,667,243	104.215	0.3281	4,072,000	JAGRWG000000000
2a4022t1	MSSA	4234	78,283	84	2,739,662	158.357	0.328	6,223,001	JAGRWF000000000
2a4023n1	MSSA	30	65,350	119	2,810,922	107.137	0.3274	4,049,446	JAGRWE000000000
2a4023p1	MSSA	30	65,350	118	2,811,110	121.371	0.3275	4,563,129	JAGRWD000000000
2a4023t1	MSSA	30	85,855	84	2,828,195	486.489	0.3273	9,146,412	JAGRWC000000000
2a4024n1	MSSA	5	84,809	78	2,778,721	245.289	0.3271	9,179,810	JAGRWB000000000
2a4026n1	MSSA	30	101,596	59	2,833,877	560.695	0.3273	10,541,369	JAGRWA000000000
2a4033n1	MSSA	U	7,486	603	2,694,337	25.919	0.3276	999,908	JAGRVZ000000000
2a4034n1	MSSA	6	112,046	62	2,798,572	645.84	0.3275	11,997,034	JAGRVY000000000
2a4039t1	MSSA	97	49,252	92	2,691,456	127.338	0.3273	4,626,272	JAGRVX000000000
2a4042n1	MSSA	30	65,287	133	2,797,542	109.999	0.329	4,210,149	JAGRVW000000000
2a4047n1	MSSA	8	145,241	59	2,714,836	709.128	0.3263	12,936,127	JAGRVV000000000
2a4060n1	MSSA	30^*d*^	66,081	99	2,758,104	124.841	0.3275	4,590,729	JAGRVU000000000
2a4060p1	MSSA	30^*d*^	65,237	112	2,794,717	99.698	0.3277	3,753,463	JAGRVT000000000
2a4060t1	MSSA	30^*d*^	65,237	111	2,795,489	123.422	0.3277	4,660,746	JAGRVS000000000
2a4064n1	MSSA	8^*c*^^,^^*d*^	46,659	110	2,709,980	23.515	0.3314	992,389	JAGRVR000000000
2a5044t1	MSSA	5	72,413	79	2,711,121	77.622	0.3272	2,830,095	JAGRVQ000000000
2a5058n1	MSSA	15^*c*^^,^^*d*^	8,147	559	2,647,664	21.36	0.3269	795,963	JAGRVP000000000
2a5062p1	MSSA	97	49,252	91	2,689,012	130.171	0.3274	4,694,918	JAGRVO000000000
2a5062t1	MSSA	97	48,945	97	2,689,173	98.019	0.3274	3,584,907	JAGRVN000000000
2a5064n1	MSSA	5	78,282	83	2,696,476	285.543	0.3276	10,285,666	JAGRVM000000000
2a5066n1	MSSA	5^*d*^	72,401	82	2,689,836	75.379	0.3305	3,126,770	JAGRVL000000000
2a5067n1	MSSA	45	82,767	67	2,684,802	31.373	0.3305	1,280,727	JAGRVK000000000
2a5067t1	MSSA	45	17,272	257	2,680,081	22.812	0.3277	893,379	JAGRVJ000000000
2b2003t1	MSSA	5^*d*^	75,537	81	2,717,214	108.973	0.327	3,944,772	JAGRVI000000000
2b2004t1	MSSA	30	49,583	144	2,775,085	26.806	0.3326	1,435,359	JAGRVH000000000
2b2015n1	MSSA	30	65,287	114	2,812,352	88.929	0.3274	3,386,929	JAGRVG000000000
2b2015t1	MSSA	5	79,876	83	2,729,458	132.229	0.3269	4,831,563	JAGRVF000000000
2b2024t1	MSSA	30	108,461	63	2,795,850	495.985	0.3272	9,194,168	JAGRVE000000000
2b2027n1	MSSA	6	80,528	85	2,703,882	107.351	0.3281	4,548,896	JAGRVD000000000
2b2027p1	MSSA	6	82,878	88	2,702,738	129.609	0.3272	4,679,989	JAGRVC000000000
2b2027t1	MSSA	6	70,596	87	2,702,646	119.361	0.3271	4,333,009	JAGRVB000000000
2b2036t1	MSSA	U	77,767	80	2,685,105	40.066	0.3286	1,720,471	JAGRVA000000000
2b2037t1	MSSA	15	41,150	119	2,641,280	30.562	0.3293	1,358,863	JAGRUZ000000000
2b2039n1	MSSA	5^*d*^	88,186	80	2,709,869	130.281	0.3273	4,933,848	JAGRUY000000000
2b2041t1	MSSA	291	50,411	99	2,690,148	26.866	0.3318	1,326,414	JAGRUX000000000
2b2042t1	MSSA	188	74,109	77	2,696,551	348.13	0.3272	12,530,947	JAGRUW000000000
2b2044n1	MSSA	5	14,293	334	2,686,667	23.799	0.3266	864,275	JAGRUV000000000
2b2044p1	MSSA	5	62,109	84	2,697,345	162.483	0.3271	6,135,231	JAGRUU000000000
2b2044t1	MSSA	5	61,502	85	2,693,572	42.598	0.3279	1,584,996	JAGRUT000000000
2b2045n1	MSSA	6	63,783	88	2,757,627	132.062	0.3274	4,871,947	JAGRUS000000000
2b2050p1	MSSA	582	61,484	104	2,683,757	46.385	0.3287	2,192,845	JAGRUR000000000
2b2050t1	MSSA	582	109,343	53	2,700,985	757.844	0.3274	13,594,931	JAGRUQ000000000
2b2054t1	MSSA	87	67,173	90	2,690,298	186.92	0.3277	6,711,788	JAGRUP000000000
2b2056n1	MSSA	27	74,666	76	2,693,190	156.334	0.3269	5,676,539	JAGRUO000000000
2b2057t1	MSSA	398	71,326	86	2,692,776	103.615	0.3285	3,724,537	JAGRUN000000000
2b2058t1	MSSA	45	45,685	122	2,695,612	18.384	0.3372	907,536	JAGRUM000000000
2b2068t1	MSSA	97	49,252	96	2,689,283	112.84	0.3275	4,156,542	JAGRUL000000000
2b2071n1	MSSA	5	72,413	91	2,771,743	140.065	0.3271	5,180,223	JAGRUK000000000
2b3002t1	MSSA	8	88,190	80	2,752,615	141.534	0.3271	5,214,623	JAGRUJ000000000
2b3005t1	MSSA	45	83,096	62	2,659,432	141.283	0.3284	5,063,498	JAGRUI000000000
2b3015n1	MSSA	45	62,499	75	2,720,112	143.28	0.3284	5,192,706	JAGRUH000000000
2b3020t1	MSSA	8	59,267	99	2,721,444	53.859	0.327	2,178,330	JAGRUG000000000
2b3028t1	IV(2B)	5	55,008	89	2,770,872	31.635	0.3299	1,587,011	JAGRUF000000000
2b3034n1	MSSA	5	72,469	88	2,746,936	150.28	0.3279	5,513,845	JAGRUE000000000
2b3034t1	MSSA	5^*d*^	14,848	345	2,740,670	21.742	0.3272	837,284	JAGRUD000000000
2b3045n1	MSSA	45	62,492	81	2,778,381	138.767	0.3291	5,642,311	JAGRUC000000000
2b3045t1	MSSA	45	72,175	75	2,694,988	27.714	0.3326	1,080,157	JAGRUB000000000
2b3048n1	MSSA	30	65,917	94	2,799,166	136.285	0.3272	5,093,361	JAGRUA000000000
2b3048t1	MSSA	30	65,917	94	2,797,777	153.266	0.3271	5,791,938	JAGRTZ000000000
2b3049t1	MSSA	4302	8,205	577	2,691,828	13.78	0.3275	898,469	JAGRTY000000000
2b3067n1	MSSA	U	171,989	45	2,725,736	505.288	0.3278	9,121,432	JAGRTX000000000
2b3070n1	MSSA	15^*c*^^,^^*d*^	51,967	104	2,662,144	140.3	0.3269	5,029,884	JAGRTW000000000
2b3070p1	MSSA	15^*c*^^,^^*d*^	55,659	101	2,661,648	122.61	0.327	4,364,407	JAGRTV000000000
2b3070t1	MSSA	15^*c*^^,^^*d*^	55,633	104	2,660,629	120.194	0.327	4,367,431	JAGRTU000000000
2b3074n1	MSSA	30^*d*^	8,211	558	2,792,658	11.183	0.3269	602,805	JAGRTT000000000
2b3074t1	MSSA	30	53,121	137	2,839,270	46.81	0.3335	2,404,170	JAGRTS000000000
2b4022n1	MSSA	5	72,469	88	2,746,346	127.745	0.3281	4,721,229	JAGRTR000000000
2b4022t1	MSSA	97	48,755	98	2,691,791	101.319	0.3293	4,067,469	JAGRTQ000000000
2b4024n1	MSSA	188	73,065	88	2,700,452	119.163	0.3271	4,323,271	JAGRTP000000000
2b4024t1	MSSA	188	69,232	86	2,700,751	125.062	0.3271	4,516,234	JAGRTO000000000
2b4027n1	MSSA	121	65,074	89	2,776,827	142.093	0.3276	5,332,504	JAGRTN000000000
2b4027t1	MSSA	121	52,515	89	2,777,144	114.892	0.3276	4,266,895	JAGRTM000000000
2b4034p1	MSSA	6	71,551	101	2,781,861	54.936	0.3285	2,513,929	JAGRTL000000000
2b4034t1	MSSA	5^*d*^	70,571	83	2,688,692	59.111	0.3321	2,358,983	JAGRTK000000000
2b4042t1	MSSA	45	23,425	211	2,761,436	17.722	0.3294	838,633	JAGRTJ000000000
2b4047n1	MSSA	30	61,920	100	2,819,896	238.065	0.3276	8,979,682	JAGRTI000000000
2b4049p1	MSSA	188	75,493	81	2,703,129	159.8	0.3275	7,035,277	JAGRTH000000000
2b5018n1	MSSA	6	62,625	114	2,771,464	117.266	0.3277	4,356,217	JAGRTG000000000
2b5066n1	MSSA	30	66,929	89	2,777,231	83.516	0.3286	3,419,491	JAGRTF000000000
2c3015n1	MSSA	30	61,920	119	2,823,029	135.415	0.3277	5,124,348	JAGRTE000000000
2c3025n1	MSSA	30	61,993	117	2,813,001	115.522	0.3275	4,386,941	JAGRTD000000000
2c3025t1	MSSA	30	61,993	120	2,812,900	100.235	0.3274	3,891,931	JAGRTC000000000
2c3030n1	MSSA	1821	53,240	117	2,724,369	136.603	0.3256	4,973,804	JAGRTB000000000
2c3041t1	MSSA	8	52,751	87	2,787,640	90.25	0.3268	3,434,811	JAGRTA000000000
2c3057p1	MSSA	106	80,824	87	2,738,220	142.275	0.3277	5,250,575	JAGRSZ000000000
2c4024n1	MSSA^*c*^	944	99,112	57	2,745,750	136.994	0.3268	5,005,694	JAGRSY000000000
2c4024t1	MSSA^*c*^	944	99,671	55	2,745,833	137.143	0.3268	5,006,024	JAGRSX000000000
2c4027n1	MSSA	30	62,983	111	2,774,672	102.927	0.3275	3,859,238	JAGRSW000000000
2c4027t1	MSSA	30	65,287	110	2,775,568	145.036	0.3275	5,383,842	JAGRSV000000000
2c4042t1	MSSA	45	63,886	84	2,782,623	102.745	0.3283	3,897,755	JAGRSU000000000
2c4044n1	MSSA	97	50,474	92	2,686,005	132.317	0.3275	4,829,497	JAGRST000000000
2c4064t1	MSSA	188	55,148	109	2,791,340	59.428	0.3277	2,485,190	JAGRSS000000000
2c5016t1	MSSA	45^*d*^	72,744	66	2,723,492	40.083	0.3285	1,458,565	JAGRSR000000000
2c5053n1	MSSA	182^*d*^	207,252	47	2,784,661	437.821	0.3268	8,103,136	JAGRSQ000000000
2c5053p1	MSSA^*c*^	182^*d*^	87,946	85	2,796,278	194.882	0.3267	7,266,336	JAGRSP000000000
2c5058n1	MSSA^*c*^	182^*d*^	97,369	56	2,771,715	109.234	0.327	4,037,138	JAGRSO000000000
2c5062n1	MSSA	8	86,210	80	2,761,633	114.358	0.327	4,236,363	JAGRSN000000000
2c5062t1	MSSA	8	86,210	76	2,760,480	130.597	0.327	4,864,229	JAGRSM000000000
2c6023n1	MSSA	5	62,107	79	2,709,038	144.606	0.3282	5,226,099	JAGRSL000000000
2c6023p1	MSSA	5	62,107	79	2,709,408	124.925	0.3282	4,523,923	JAGRSK000000000
2c6027n1	MSSA	20	55,344	96	2,710,726	116.982	0.3271	4,245,848	JAGRSJ000000000
2d4008n1	MSSA	30^*d*^	59,006	113	2,825,854	133.957	0.3276	5,051,298	JAGRSI000000000
2d4019n1	MSSA	5	72,525	82	2,668,760	144.552	0.3277	5,219,833	JAGRSH000000000
2d4019t1	MSSA	5	106,868	51	2,710,442	402.459	0.327	7,215,947	JAGRSG000000000
2d4026p1	MSSA	582	48,670	113	2,712,976	146.263	0.3268	5,336,331	JAGRSF000000000
2d4026t1	MSSA	8	86,471	75	2,716,950	122.055	0.3267	4,436,880	JAGRSE000000000
2d4044n1	MSSA	45	72,743	65	2,655,305	164.239	0.3281	5,812,401	JAGRSD000000000
2d4047t1	MSSA	8	36,286	158	2,749,649	22.197	0.334	920,513	JAGRSC000000000
2d4064t1	MSSA	121	50,853	109	2,714,807	21.989	0.3284	1,039,925	JAGRSB000000000
2d4067n1	MSSA	97	48,938	97	2,690,260	43.313	0.3341	1,699,501	JAGRSA000000000
2d4072n1	MSSA	30	66,076	87	2,726,537	206.714	0.3275	7,546,788	JAGRRZ000000000
2d5017n1	MSSA	15	72,774	68	2,754,631	94.439	0.3279	3,519,597	JAGRRY000000000
2d5044n1	MSSA	97	44,317	107	2,633,073	27.209	0.3311	1,449,531	JAGRRX000000000
2d5058n1	MSSA	508	76,890	69	2,706,767	130.345	0.3279	4,710,403	JAGRRW000000000
2d5058t1	MSSA	508	75,567	71	2,705,362	46.968	0.3308	1,952,900	JAGRRV000000000
2d5066t1	MSSA	30	65,350	97	2,821,246	117.842	0.3276	4,484,517	JAGRRU000000000
2e5017n1	MSSA	15	50,905	116	2,722,037	123.819	0.3272	4,540,994	JAGRRT000000000
2e5018t1	MSSA	25^*c*^^,^^*d*^	87,659	71	2,705,734	138.923	0.3269	5,016,178	JAGRRS000000000
2e5053n1	MSSA	30	52,179	134	2,776,357	29.303	0.3298	1,397,474	JAGRRR000000000
2e5053p1	MSSA	30	87,946	85	2,796,278	554.642	0.3272	10,281,371	JAGRRQ000000000
2e5053t1	MSSA	30	52,923	138	2,814,956	30.356	0.3306	1,652,949	JAGRRP000000000
2e5062n1	MSSA	30	63,699	88	2,767,914	102.402	0.3276	3,846,202	JAGRRO000000000
2e6023n1	MSSA	72	83,834	76	2,660,285	393.925	0.3274	14,017,959	JAGRRN000000000
2e6027t1	MSSA	U	60,584	78	2,754,672	110.934	0.3276	4,081,324	JAGRRM000000000
2f6027n1	MSSA	6	62,626	90	2,698,531	116.987	0.3273	4,256,944	JAGRRL000000000
A	MSSA	30	110,216	83	2,782,411	149.833	0.3276	6,177,316	JAGRRK000000000

a For each isolate, the individual person is indicated by the first 6 characters, and the source is indicated by n (nares), p (palm), or t (throat) as the 8th character.

b U, unknown.

c Alleles with less than 100% coverages found.

d Alleles with less than 100% identity found.

### Data availability.

The genome sequences are available in GenBank under BioProject accession no. PRJNA660486.

## References

[1] Chambers HF, DeLeo FR. 2009. Waves of resistance: Staphylococcus aureus in the antibiotic era. Nat Rev Microbiol 7:629–641. doi:10.1038/nrmicro2200. 19680247 PMC2871281

[2] Williams REO. 1963. Healthy carriage of Staphylococcus aureus: its prevalence and importance. Bacteriol Rev 27:56–71. doi:10.1128/br.27.1.56-71.1963. 14000926 PMC441169

[3] Kluytmans J, van Belkum A, Verbrugh H. 1997. Nasal carriage of Staphylococcus aureus: epidemiology, underlying mechanisms, and associated risks. Clin Microbiol Rev 10:505–520. doi:10.1128/CMR.10.3.505. 9227864 PMC172932

[4] Wertheim HFL, Melles DC, Vos MC, van Leeuwen W, van Belkum A, Verbrugh HA, Nouwen JL. 2005. The role of nasal carriage in Staphylococcus aureus infections. Lancet Infect Dis 5:751–762. doi:10.1016/S1473-3099(05)70295-4. 16310147

[5] von Eiff C, Becker K, Machka K, Stammer H, Peters G. 2001. Nasal carriage as a source of Staphylococcus aureus bacteremia. N Engl J Med 344:11–16. doi:10.1056/NEJM200101043440102. 11136954

[6] Lina G, Piemont Y, Godail-Gamot F, Bes M, Peter M-O, Gauduchon V, Vandenesch F, Etienne J. 1999. Involvement of Panton-Valentine leukocidin–producing Staphylococcus aureus in primary skin infections and pneumonia. Clin Infect Dis 29:1128–1132. doi:10.1086/313461. 10524952

[7] Pannaraj PS, Hulten KG, Gonzalez BE, Mason EO, Jr, Kaplan SL. 2006. Infective pyomyositis and myositis in children in the era of community‐acquired, methicillin‐resistant *Staphylococcus aureus* infection. Clin Infect Dis 43:953–960. doi:10.1086/507637. 16983604

[8] Gorak EJ, Yamada SM, Brown JD. 1999. Community-acquired methicillin-resistant Staphylococcus aureus in hospitalized adults and children without known risk factors. Clin Infect Dis 29:797–800. doi:10.1086/520437. 10589891

[9] Gonzalez BE, Rueda AM, Shelburne SA, III, Musher DM, Hamill RJ, Hulten KG. 2006. Community-associated strains of methicillin-resistant Staphylococccus aureus as the cause of healthcare-associated infection. Infect Control Hosp Epidemiol 27:1051–1056. doi:10.1086/507923. 17006811

[10] Kennedy LA, Gill JA, Schultz ME, Irmler M, Gordin FM. 2010. Inside-out: the changing epidemiology of methicillin-resistant Staphylococcus aureus. Infect Control Hosp Epidemiol 31:983–985. doi:10.1086/655837. 20677980

[11] David MZ, Cadilla A, Boyle-Vavra S, Daum RS. 2014. Replacement of HA-MRSA by CA-MRSA infections at an academic medical center in the Midwestern United States, 2004–5 to 2008. PLoS One 9:e92760. doi:10.1371/journal.pone.0092760. 24755631 PMC3995643

[12] Gorwitz RJ, Kruszon‐Moran D, McAllister SK, McQuillan G, McDougal LK, Fosheim GE, Jensen BJ, Killgore G, Tenover FC, Kuehnert MJ. 2008. Changes in the prevalence of nasal colonization with Staphylococcus aureus in the United States, 2001–2004. J Infect Dis 197:1226–1234. doi:10.1086/533494. 18422434

[13] Wertheim HFL, Vos MC, Ott A, van Belkum A, Voss A, Kluytmans JAJW, van Keulen PHJ, Vandenbroucke-Grauls CMJE, Meester MHM, Verbrugh HA. 2004. Risk and outcome of nosocomial Staphylococcus aureus bacteraemia in nasal carriers versus non-carriers. Lancet 364:703–705. doi:10.1016/S0140-6736(04)16897-9. 15325835

[14] Kluytmans JAJW, Mouton JW, Ijzerman EPF, Vandenbroucke-Grauls CMJE, Maat AWPM, Wagenvoort JHT, Verbrugh HA. 1995. Nasal carriage of Staphylococcus aureus as a major risk factor for wound infections after cardiac surgery. J Infect Dis 171:216–219. doi:10.1093/infdis/171.1.216. 7798667

[15] Kalmeijer MD, van Nieuwland-Bollen E, Bogaers-Hofman D, de Baere GAJ, Kluytmans JAJW. 2000. Nasal carriage of Staphylococcus aureus: is a major risk factor for surgical-site infections in orthopedic surgery. Infect Control Hosp Epidemiol 21:319–323. doi:10.1086/501763. 10823564

[16] Huang SS, Platt R. 2003. Risk of methicillin‐resistant Staphylococcus aureus infection after previous infection or colonization. Clin Infect Dis 36:281–285. doi:10.1086/345955. 12539068

[17] Graham PL, III, Lin SX, Larson EL. 2006. A US population-based survey of Staphylococcus aureus colonization. Ann Intern Med 144:318–325. doi:10.7326/0003-4819-144-5-200603070-00006. 16520472

[18] Barger SD, Lininger MR, Trotter RT, Jr, Wayment HA, Mbegbu M, Kyman S, Pearson T. 2020. Educational attainment and *Staphylococcus aureus* colonization in a Hispanic border community: testing fundamental cause theory. mSphere 5:e00623-20. doi:10.1128/mSphere.00623-20. 32999080 PMC7529436

[19] Pearson T, Barger SD, Lininger M, Wayment H, Hepp C, Villa F, Tucker-Morgan K, Kyman S, Cabrera M, Hurtado K, Menard A, Fulbright K, Wood C, Mbegbu M, Zambrano Y, Fletcher A, Medina-Rodriguez S, Manone M, Aguirre A, Milner T, Trotter RT, Jr. 2019. Health disparities in Staphylococcus aureus transmission and carriage in a border region of the United States based on cultural differences in social relationships: protocol for a survey study. JMIR Res Protoc 8:e14853. doi:10.2196/14853. 31573953 PMC6789426

[20] Bankevich A, Nurk S, Antipov D, Gurevich AA, Dvorkin M, Kulikov AS, Lesin VM, Nikolenko SI, Pham S, Prjibelski AD, Pyshkin AV, Sirotkin AV, Vyahhi N, Tesler G, Alekseyev MA, Pevzner PA. 2012. SPAdes: a new genome assembly algorithm and its applications to single-cell sequencing. J Comput Biol 19:455–477. doi:10.1089/cmb.2012.0021. 22506599 PMC3342519

[21] Bushnell B. 2014. BBMap. https://sourceforge.net/projects/bbmap/.

[22] Tatusova T, DiCuccio M, Badretdin A, Chetvernin V, Nawrocki EP, Zaslavsky L, Lomsadze A, Pruitt KD, Borodovsky M, Ostell J. 2016. NCBI Prokaryotic Genome Annotation Pipeline. Nucleic Acids Res 44:6614–6624. doi:10.1093/nar/gkw569. 27342282 PMC5001611

[23] Center for Genomic Epidemiology. http://www.genomicepidemiology.org/.

